# St. George's Hospital

**Published:** 1893-11-18

**Authors:** 


### HOSPITAL MEETINGS.

### ST. GEORGE'S HOSPITAL.

The quarterly meeting of the Governors of St. George's Hospital, held on Friday of last week, was brief and harmonious; nevertheless the business had an importance of its own in relation to the efficiency of the hospital. In the company of about a score of governors present the army and navy were exceptionally well represented. Colonel Haygarth occupied the chair, supported by Admiral Sir W. H. Stewart, Major-General Gifford, and Captain Neal. The proposal to erect a new steam boiler and boiler-house for the hospital was first considered. It will cost £3,500 to make these and other improvements, and on the motion of the Treasurer, Mr. J. R. Mosse, this expenditure was sanctioned. The recom mendation of the Duke of Westminster, the ground landlord, that the new boiler-house should consume its own smoke, will be carried out. Mr. R. Brudenell Carter—now retiring from active duty—who has been for twenty-three years on the surgical staff of the hospital, was appointed consulting ophthalmic surgeon in token of appreciation of his great services to the institution. The sanction for a new arrangement in regard to the administration of anæsthetics at the hospital was asked. Hitherto, it appears, the Surgical Registrar has been anæsthetist, receiving in recognition of his services in this connection an addition to his salary of £20 a year. The new proposal, which was carried, is that henceforth two qualified practitioners will be appointed anæsthetists. The senior anæsthetist will receive a salary of £50 per annum, and the junior £20. At this point in the proceedings the Secretary, Mr. Charles L. Todd, left the room. The reason for this became obvious when Mr. Mosse, the Treasurer, rose to pass a high encomium upon Mr. Todd's services as secretary and superintendent for the last thirty years, and to move that, in recognition of his arduous and faithful services, his salary be increased by £50 per annum. The suggestion met with hearty approval, and was adopted amid applause. The effect of this resolution is to raise Mr. Todd's salary from £450 to £500—a most modest remuneration for services such as Mr. Todd's, which we hope to see speedily and materially increased. A list was submitted of subscribers who had been removed by death—among them Sir Andrew Clark—and of persons who had ceased to subscribe. It was elicited that the sum represented by new subscribers came far short of the subscriptions that had to be written off. Mr. Mosse stated that sometimes the heirs of deceased subscribers continued the subscription, but this was by no means the general rule. In reply to a question, the Treasurer stated that the expenditure of the hospital might be reckoned at about 30s. per patient per week. Another gentleman asked what the term "domestic" expenditure in the accounts meant? and the Secretary gave information as to the items covered by the term in the scheme of accounts recommended by the Hospital Sunday Fund, which is followed at St. George's.

